# Annotations

**Published:** 1891-07-25

**Authors:** 


### Annotations.

**Music and Medicine.**

The *Lancet* gives a very cautious reply to a suggestion as to the use of music as a medical treatment. "Five years ago," says the *Lancet's* correspondent, "I had an opportunity of trying the effect of dreamy music upon a lady of great intellectual power, who retained, too, her faculties at the ripe age of eighty-six. About seven minutes were occupied by the music, and before its last notes were heard my revered friend, the Viscountess Combermere, had closed her eyes and was napping." This story reminds us of another told by the late Dean Ramsey in his *Reminiscences of Scottish Life and Character*. A certain country laird was taken ill with some affection which produced marked sleeplessness. All sorts of remedies for the insomnia were tried, but tried in vain. The laird had a son who was what is called in Scotland "daft," that is, he was somewhat weak in the upper storey. When the other members of the laird's family were in a state bordering on distraction, the lad whom nobody thought of taking into consultation, suddenly burst out with, "Feyther aye sleeps i' the kirk." The suggestion of getting a minister to preach to the sleepless man was acted upon immediately, and with the best results. Hardly had the reverend divine got well into the second head of his discourse, before the patient was sound asleep and snoring like the drone of a bag-pipe. The peculiar monotony of the preacher's voice had acted as an irresistible soporific. It is a common experience that the monotonous reading of a book, or the measured cadences of quiet singing, are often of great value in the soothing of the nervous system. There is perhaps no need to institute an order of bed-room musical performers. But it might be well if nurses were taught to chant a little, and were to learn suitable music for the bedside. Young ladies, too, and even matrons, would be all the better if, in the course of their ordinary education, they had a little instruction in music of a sleep-inducing kind. There is manifestly a field for the musical composer also, as well as for the nurse and the young lady. The suggestion brought forward in the *Lancet*, is good, and provided always that everything is done under the guidance of the physician in charge, there is every reason to hope that much suffering may be mitigated, and comfort and health greatly promoted.

**Why are Sixpenny Doctors Objectionable?**

A correspondent writes: "I cannot see why you have so strong an objection to sixpenny doctors." The present writer, a doctor himself, objects to sixpenny doctors, chiefly because it is practically impossible for them to be honest. He himself, a good many years ago, spent six or eight months as an experimental partner, with a "shilling" doctor, that is a man who charged a shilling a quarter for what is now offered for sixpence. He found it altogether impossible to give the necessary time for skilled treatment to his shilling patients, and equally impossible to give them the kind of medicines which his best judgment dictated. In addition to this personal experience he critically watched the practice of his temporary partner and of his three assistants. After six months of daily observation and experience he came to the conclusion that medical practice as conducted at that particular dispensary was almost of necessity rank knavery. For himself he resolved that rather than cheat and swindle the poor people by hurried attendance and cheap and useless physic, he would quit the medical profession altogether. He has since seen many facts and circumstances which have confirmed that judgment. So far from sixpence a quarter being sufficient remuneration to enable a doctor to do even the minimum of justice to both his patients and himself, it is the fact that six times sixpence is not more than enough. In the case of men who are at work, and in the full vigour of life, five shillings a year may perhaps be considered sufficient to give the doctor a very modest remuneration if he have a sufficiently large club composed of such men. But women and children, more particularly in towns, ordinarily require three or four times as much attendance and medicine as men. At the particular shilling dispensary of which the writer had a prolonged experience, it was no uncommon thing for thirty or forty women and children to be seen and prescribed for every day. For every man who came there were at least twenty women and children. Doctors never seem to work these things out in a business-like way. They have no head for business as merchants and manufacturers have. If the principles of dispensary practice were applied to trade, business men would be ruined by hundreds. Medical men use their clubs as the means of getting into better practice; and when the club fees are so low as sixpence a quarter the general results to the patients are that they are the blind and helpless victims of the doctor's haste and poverty. It would be just as easy for a man to climb to the moon without a staircase as it is for a medical practitioner to give really skilled attendance and suitable medicine to children or women for sixpence a quarter. Both these things are impossible, and the one is as impossible as the other. Sixpenny doctors bring both the art of medicine and the character of its professors into merited and general contempt. That being undoubtedly the case, every doctor who respects himself and his profession is bound to object to them, and to try to stamp them out on all suitable occasions.

**The Mathews Duncan Memorial.**

The great world does not honour medical men very greatly. It is all the more necessary, therefore, that the medical profession itself should strive to do justice to those of its members who have been conspicuous for personal and professional worth. Dr. Mathews Duncan, the late obstetric physician to St. Bartholomew's Hospital, was a man of much originality of mind, of the highest professional capacity, and of uncommon integrity of character. The particular specialty to which he devoted himself is not one which requires any very extraordinary ability, or which tends to greatly develop the intellect. What, however, it does demand is good powers of observation, sound judgment, kindness, and the most self-denying honesty. Dr. Mathews Duncan fulfilled all these conditions in an eminent degree. He was a most painstaking observer of the actual facts of every case brought before him. His judgment, informed by extensive reading and large experience, was sound and clear. He did not fear to differ from other men, although he always took care to rest his differing decision on facts. He was most kind and considerate to all his patients, rich and poor alike. As to manner, he was a North-country Scotchman; and sometimes Scotchmen of the North country give to the milder-mannered Southerner the impression of brusque off-handedness. But Dr. Duncan could not have been unkind if he had tried. The great characteristic, however, of the man was his plain and simple honesty. He would not depart from the truth by one hair's breadth. His specialty, as we have pointed out in these pages before, lends itself in a very peculiar way to all sorts of the meanest and most miserable arts of knavery. If a man, after practising as an obstetric physician for forty years, still retains a true and honest simplicity, he has shown a strength of character which many a martyr has probably not possessed. St. Bartholomew's Hospital, and many leading physicians, surgeons, scientific men, and others who have nothing to do with the hospital, have very distinctly and fully recognised Dr. Duncan's rare worth. The hospital and those various outsiders are about to establish a Mathews Duncan memorial prize, and to place a bust of the dead man in the college library. In the absence of any more worthy memorial, that which is proposed is fitting. As we have said, the great world cares little for doctors; knows nothing about them, in fact. For its own sake we wish the case were otherwise. Stern simplicity allied to strong capacity, and devoted in the most strenuous way to a comparatively second-rate branch of medical practice, affords an example of high devotion to lowly duty which could not but be of great service to all kinds of men.

